# Anti-aging effect and gene expression profiling of dung beetle glycosaminoglycan in aged rats

**DOI:** 10.1186/s40824-017-0091-9

**Published:** 2017-04-21

**Authors:** Mi Young Ahn, Ban Ji Kim, Ha Jeong Kim, Jae Sam Hwang, Yi-Sook Jung, Kun-Koo Park

**Affiliations:** 1 Department of Agricultural Biology, National Academy of Agricultural Science, Rural Development Administration (RDA), Wanju-Gun, 55365 South Korea; 2 College of Pharmacy, Ajou University, Suwon, 442-749 South Korea; 3 Pharmacogenechips Inc., Chuncheon, 200-160 South Korea

**Keywords:** Anti-aging effect, Dung beetle (*Catharsius molossus*), Queen of *B. ignitus*, Glycosaminoglycan

## Abstract

**Background:**

This study aimed to evaluate the anti-aging effect of a newly prepared insect-derived compound, dung beetle glycosaminoglycan (GAG), given intraperitoneally to old SD rats as part of their diet for 1 month. Insect GAG administration was found to be related to a reduction in oxidative damage, hepato-cellular biomarker levels, protein carbonyl content, and malondialdehyde concentration. The anti-aging-related molecular genetic mechanisms of dung beetle GAG are not yet fully elucidated.

**Results:**

*Catharsius molossus* (a type of dung beetle) GAG (CaG) possessed anti-aging activities; it reduced the serum level of creatinine kinase, had aortic vasorelaxant activities and cardioprotective actions, and maintained a normal glucose level in treated rats. Microarray analysis was performed with a rat 30 K cDNA clone set array to identify the gene-expression profiles of 14-month-old SD rats treated with dung beetle glycosaminoglycan 5 mg/kg (CaG5) over a 1-month period, which was done to investigate its anti-aging effect as compared to that of either *Bombus ignitus* (a type of bumblebee) queen GAG 5 mg/kg (IQG5) or chondroitin sulfate 10 mg/kg. CaG5 and IQG5 had marked anti-inflammatory effects, bringing about inhibition of free fatty acid, uric acid, sGPT, IL-1 beta, and CK values. In addition, anticoagulant and antithrombotic effects were seen: the concentration of factor 1 (fibrinogen) was increased in CaG- treated rat plasma. The CaG5-treated rat group, compared to the control, displayed upregulation of 131 genes, including lipocalin 2 (Lbp) and a serine peptidase inhibitor, Kaszal type3 (Spink3), and 64 downregulated genes, including lysyl oxidase (Lox), serine dehydratase (sds), and retinol saturase (Retsat).

**Conclusion:**

Our data suggest that dung beetle glycosaminoglycan may be a helpful treatment for aged rats, which indicates its potential as a therapeutic biomaterial for aging.

## Background

Aging is associated with an increased risk of cardiovascular disease and death [1]. Here, we show that intraperitoneal supplementation with natural insect GAGs exerts cardioprotective effects via vasoconstriction, by reducing the serum level of creatinine kinase in old rats. In fact, although GAGs constitute a minor portion of native tissues, they play a crucial role in various physiological processes [2]. There are many reports that GAGs are related to age; they have been implicated in such processes as degeneration of cervical intervertebral discs [3, 4], age-related diseases that impair bone healing [5], and prevention of cancer [6]. They have been shown to be associated with age-related changes in matrix components and the onset of diseases of aging, especially cardiovascular pathologies, and are mostly involved in the age-dependence of biological functions and the limitation of longevity [7]. Aging is associated with alterations in the structure of heparin sulfate on the surface of outgrowth endothelial cells. Such changes modulate the migration, homing, and engraftment capacity of these repair cells, thereby contributing to the progression of endothelial dysfunction and age-related vascular pathologies [8]. Edible and medicinal insects have been employed, by way of purification, extraction, and identification of potent active constituents, as functional foods or drugs. Extracts of the dung beetle *Catharsius molossus*, which is found in China, displayed marked fibrinolytic activity and anti-oxidative and anti-hyperglycemic effects on rats fed a high fat diet [9, 10]. The protein carbonyl content of blood and malondialdehyde concentration of liver tissue treated with dung beetle ethanol or acetone extract over a 1-month period, were significantly decreased compared to the controls [10]. Recently, a Korean dung beetle peptide, LLCIALRKK-NH_2_, which is a 9-mer peptide derived from *Copris tripartitus* Coprisin, was found to exert bactericidal activity against E. coli and antimicrobial activity by causing severe DNA damage, which induces apoptosis-like death [11]. An apicultural product, *B. ignitus* queen (BIQ) alcohol extract has the highest potential efficacy amongst all bumble bee extracts tested for treating inflammation in SD rats, as it significantly reduced paw edema [12]. So, we prepared GAGs derived from the greatest acquirable number of *C. molossus* or *B. ignitus* queens to test their anti-aging properties. Aging involves a progressive decline in the physiological capacity of an organism, and is manifested by accumulated alterations and destabilization at the whole system level [13]. Aging is also associated with a differential gene expression pattern indicative of a decreased stress response amongst metabolic and biosynthetic genes [14]. In particular, an age- or obesity-related increase in visceral adipose tissue is usually accompanied by low-grade chronic inflammation, which has been postulated as a cause of various metabolic diseases including cancer, cardiovascular diseases, and, most prominently, type two diabetes [15]. These insect GAGs can be used in a fast developing field with the prospect of utilizing tissue engineering and biomaterials as novel therapies [16].

In this study, we report that GAG from dung beetles and BIQ displayed anti-aging properties and, thus, these compounds hold great promises for use as anti-aging agents. Also, we demonstrate the potential value of *C. molossus* dung beetle glycosaminoglycan in lessening the deleterious aspects of aging, as measured both in serum and in the gene expression patterns of 14-month-old SD rats following treatment for 1 month.

## Methods

### Materials

#### Preparation of insect glycosaminoglycan

Dried *C. molossus* was purchased at a local market in China; bumble bee (queen of *Bombus ignitus*) was reared and freeze-dried, in the Department of Agricultural Biology, National Academy of Agricultural Science, South Korea. Chondroitin sulfate and all reagents were supplied from Sigma Aldrich (St. Louis, Mo., USA).

Each of one-kilogram (1 kg) dried insect was soaked and extracted three times with ethanol by ultrasonification (Branson, Colorado, MI, USA) for 30 min. The residues separated from the alcohol extracts were defatted twice with 2 volumes of acetone. Approximately 200 g of dried, defatted and pulverized powder was suspended in 2 L of 0.05 M sodium carbonate buffer (pH 9). The suspension was incubated for 48 h at 60 °C after adding 28 ml (1.4%) of Alcalase (Sigma Aldrich, St. Louis, Mo., USA). The digestion mixture was cooled to 4 °C, and trichloroacetic acid was added to a final concentration of 5%. The sample was mixed, allowed to stand for 1 h, and then centrifuged for 30 min at 8000 × g (Hanil Science Industrial, Incheon, South Korea). Three volumes of 5% potassium acetate in ethanol were added to one volume of supernatant. After mixing, the suspension was stored overnight at 4 °C and then centrifuged. The precipitate amounting to 20 g was dissolved in 40 ml of 0.2 M NaCl and centrifuged. Cetylpyridinium chloride (5%) was added to 0.2 times the volume of the supernatant, and the precipitate was collected by centrifugation. The precipitate was dissolved in 20 ml of 2.5 M NaCl. Five volumes of ethanol were added, and the precipitate was separated by centrifugation. The precipitate was dissolved in water and dialyzed against 100 volumes of water [17], and the dialyzed crude glycosaminoglycan was freeze-dried to obtain about 1.1 g of CaG, 4.89 g of IQG as a powder. Crude GAG was loaded onto a DEAE Sephadex A-25 gel chromatography column (40 x 1.2 cm) equilibrated with 50 mM phosphate buffer (pH 7.4). The fractions were eluted using a linear sodium chloride gradient from 0 to 2.5 M NaCl in phosphate buffer at a flow rate of 20 ml/h, and the dialyzed glycan was freeze-dried to pure GAG.

### Animals

Sprague Dawley (SD) rats (male), at 8-months of age, were supplied from Samtako Co. Ltd. (Osan, Korea). All procedures were in accordance with the NIH Guidelines for Care and Use of Laboratory Animals. All experiments were approved by the Laboratory Animals’ Ethical Committee of the National Academy of Agricultural Science, RDA, South Korea (NAAS1503) and followed national guidelines for the care and use of animals (individual housing). The rats were acclimated for 6 months under normal husbandry conditions (23 ± 2 °C, 55 ± 10% humidity and 12 h light/dark cycle) and fed a normal diet (D10001, AIN-76A rodent diet, Research Diet Inc., New Brunswick, NJ, USA) and water *ad libitum*. The 14 month old rats were segregated into 4 treatment groups of 10 rats each and distributed according to similarity in weight (680.2 ± 9.20 g). The treatments were given in PBS daily and each administrated intraperitoneally.

**Scheme 1:**
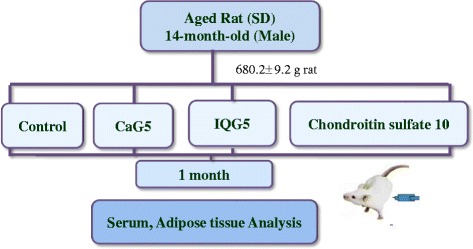
Animal experimental design

The groups were control, 5 mg/kg CaG (CaG5), 5 mg/kg IQG (IQG5), and 10 mg/kg CS (CS10) (Sigma Aldrich Co., USA), given. Each group was maintained on the normal diet (AIN-76A rodent diet, Research Diet) and sample treatment for 1 month (Scheme 1).

### Adipose tissue weights

Abdominal and epididymidal fat to-body weight ratios were determined. The measurements were after sacrifice at the end of the 1- month treatment period.

### Adipocyte density

The excised organs and adipose tissue were fixed 10% neutral formalin. After paraffin embedding, they were stained with hematoxylin and eosin, and Toluidine blue O, examined by light microscopy (Leica CTR6000, Hesse, Germany), and photographed. Adipocyte density (cells/mm^2^) was determined in treated and control tissue by toluidine blue O stain (original magnification, x400).

### Blood sampling and blood, plasma, serum assay

On four groups named CON, CaG5, IQG5, CS10 (*n* = 10), after 1 month of treatment, blood (~5 ml) was collected from the posterior vena cava under light CO_2_ inhalation and used for serum chemistry measurements. The parameters examined included phospholipid, hyaluronic acid, free fatty acid, albumin, alkaline phosphatase (ALP), glutamic oxaloacetic transaminase (GOT), glutamic pyruvic transaminase (GPT), lactic dehydrogenase (LDH), CK (creatinine phosphokinase), glucose, total cholesterol triglyceride, HDL cholesterol, LDL cholesterol, creatinine, blood urea nitrogen (BUN), total protein, uric acid and c-reactive protein (CRP). All parameters were evaluated using an autoanalyzer (Hitachi 7060 automatic clinical analyzer, Tokyo).

### Blood sampling and plasma assay for anti-coagulant activity detection

After the 1-month treatments, approximately 4 ml of plasma was collected from the posterior vena cava under light CO_2_ inhalation and centrifuged. The supernatant was used for clotting time as an anticoagulant activity test: activated partial thromboplastin time (APTT), thrombin time, prothrombin time, and factor 1 (fibrinogen) as a fibrinolytic activity assay using a fore mentioned automatic clinical analyzer according to Green Cross Lab’s manual.

### Oxidative lipid damage

To determine the oxidative lipid damage in rat hepatocytes, malondialdehyde (MDA) levels were measured with a lipid peroxidation assay using the color method involving thiobarbituric acid reactive substances (TBARS) at 535 nm [18].

### Oxidative protein damage

Liver homogenate supernatants and blood, obtained following centrifugation were used for of determination of carbonyl content. Protein oxidative stress was evaluated by measuring protein carbonyl content in the blood. Carbonyl content was determined with an enzyme-linked immunoassay according to the manufacturer’s protocol for the OxiSelect™ protein carbonyl ELISA kit (Cell Biolabs, Inc., San Diego, CA, USA). CAT activity (U/mg protein) was measured based on CAT-mediated decomposition of H_2_O_2_ [19].

### Liver homogenate preparation for oxidative enzyme detection

About four groups named CON, CaG5, IQG5, CS10, liver tissues were homogenized on ice in a 10-fold volume lysis buffer PRO-PREP™ protein extraction solution (iNtRON, Busan, Korea). The supernatant of the liver homogenate after centrifugation (800 g, 10 min) was assayed for catalase, glutathione peroxidase, glutathione s-transferase and superoxide dismutase activity according to assay manual (OxiSelect™ ELISA kit, Cell BioLabs, InC., San Diego, CA, USA).

### Cytokine IL-1β, IL-6 and IL-10 assay

On four groups named CON, CaG5, IQG5, CS10, the IL-1β or IL − 6 or IL − 10 level in insect GAG-treated rat serum was measured using commercial Elisa kits (Quantikine, R&D Systems, Inc, Minneapolis, MN, USA) according to the manufacturer’s instructions.

### RNA preparation and quantitative real-time PCR analysis

**Scheme 2:**
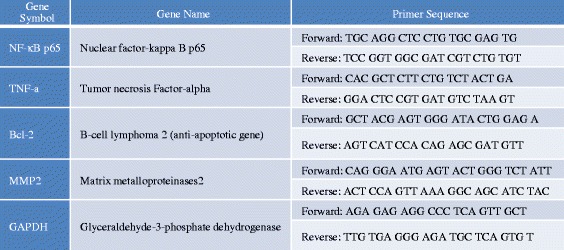
Primer sequences for amplification of genes involved in cell repair mechanism and GAPDH internal standard

Total RNA was isolated from liver using TRIzol reagent (Invitrogen, Carlsbad, CA, USA), and RNA concentration and purity were measured using a UV/Vis spectrophotometer (Beckman Coulter Co., Miami, FL, USA). Complementary DNA (cDNA) was synthesized from 1 g of total RNA using the high capacity cDNA Reverse Transcription Kit (Amersham Biosciences Co., Piscataway, NJ, USA). Real-time polymerase chain reaction (PCR) amplification was performed with Power SYBR Green Master Mix using a 7500 Real-Time PCR System (both from Applied Biosystems), according to the manufacturer’s instructions. For detection of target gene transcripts, we designed specific forward and reverse oligonucleotide primers using Beacon Designer software (PREMIER Biosoft, Palo Alto, CA, USA). The primer sequences are listed in Scheme 2. Target mRNA levels were normalized using glyceraldehyde 3-phosphate dehydrogenase (GAPDH) as an internal control to qualify the relative expression of target mRNA according to the cycling threshold method. All samples were analyzed in triplicate.

Primer sequences for amplification of genes involved in cell repair mechanism and GAPDH internal standard (Scheme 2).

### DNA microarray procedure

After histopathology analysis, microarray hybridization was performed on liver samples [20]. Total RNA was isolated from liver tissue using a Qiagen RNeasy Midi Kit (Qiagen, Valencia, CA, USA). A FairPlay™ microarray labeling kit (Stratagene, La Jolla, CA, USA) was used according to the manufacturer’s instructions. The labeled DNA was loaded onto a microarray chip. A hybridization chamber was assembled with the microarray chip, Rat Genome 230 2.0 Array (Affymetrix Inc., Santa Clara, USA), and submerged in a water bath overnight at 60 °C. The microarray chip was washed in wash buffer I, wash buffer II, and then wash buffer III for 5 or 15 min. The slide was dried by centrifuging at 500 g for 15 min and scanned with a BMS Array Scanner, applied precision Array WoRx eBiochip Reader (BioRad, Dallas, TX, USA), using the Cy3 and Cy5 channels [21].

### Isolated rat heart analysis

Male Sprague-Dawley rats weighing 350 ± 50 g were anesthetized with pentobarbital (100 mg/kg). The tail vein was injected with heparin (1000 U/kg), and then the trachea was intubated. While rats were mechanically ventilated with a rodent ventilator (Model 7025; Ugo Basile, Comerio-Varese, Italy), their hearts were perfused *in situ* with oxygenated modified Krebs-Henseleit bicarbonate buffer by retrograde aortic cannulation. The hearts were then excised and moved to a Langendorff apparatus (Hugo Sachs Electronik, March-Hugstetten, Germany), where they were perfused with oxygenated modified Krebs-Henseleit bicarbonate buffer at a constant perfusion pressure of 75 mmHg. A water-filled latex ventricle was introduced through the pulmonary vein and connected to an Isotec pressure transducer (Hugo Sachs Electronik) to measure left ventricular pressure (LVP). The hearts were allowed to equilibrate for 15 min, at which time the left ventricular end-diastolic pressure (EDP) was adjusted to 10 mmHg, and this balloon volume was maintained throughout the experiment. Then, baseline contractile function, heart rate, and coronary flow (extracorporeal electromagnetic flow probe; narco Bio-systems, Houston, TX, USA) were measured. Cardiac contractile function was calculated by subtracting LVEDP from LV peak systolic pressure (LVSP), yielding developed pressure (LVDP). After a 1 h equlibration, aortic rings were treated with vehicle (saline) or CaG (10 μg/ml, 100 μg/ml) 10 min before contraction was induced by phenylephrine 1 μM. Then, endothelium-dependent relaxation was induced by acetylcholine (0.1–100 μM) [22].

### Statistical Analysis

The means and standard error of all parameters studied were determined for each group using ANOVA test. A Student’s *t*-test was carried out to determine significant differences between control and treated groups. A *p* value <0.05 was considered significant.

## Results

### Body weight and adipose fat changes

**Fig. 1:**
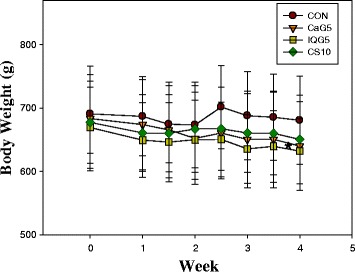
Effect of CaG on body weight in aged rat over 1 month. CaG5: Dung beetle (*C. molossus*) glycosaminoglycan 5 mg/kg. IQG5: bumblebee (queen of *B. ignitus*) glycosaminoglycan 5 mg/kg. *p < 0.05, compared with control group

**Fig. 2:**
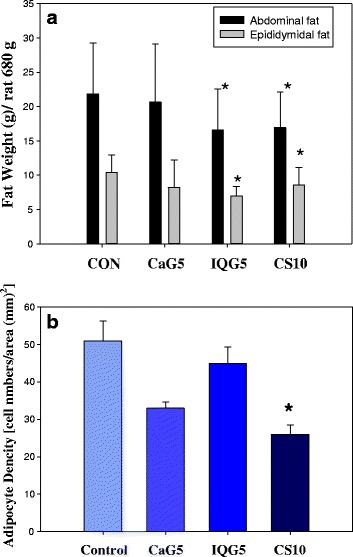
**a** Effect of dung beetle glycosaminoglycan (CaG) on adipose fat weight in aged rat. ^*^ *p* < 0.05, compared with the control group. **b** Adipocyte number in rats treated with CaG by toluidine blue O stain, (*n* = 10 per group) (*p < 0.05): significant difference vs. control group. The adipocyte cell density was counted from liver tissue toluidine blue stained depots

There were no significant differences in mean body weight between the treatment groups (Fig. 1). During the 1-month administration period, the body weights of the aged male rats were comparable in the control and CaG-, IQG-, and CS-treated groups. The mean weekly body weights are presented in Fig. 2a: CON, 685.16 ± 9.14 g; CaG5, 659.54 ± 14.21 g; IQG5, 646.50 ± 11.56 g, and CS10, 662.93 ± 7.82 g. The mean quantity of abdominal fat was significantly decreased in the IQG5 and CS10 groups compared to the control: 21.84 ± 7.46 g for the control; 20.66 ± 8.47 g for CaG5; 16.56 ± 5.97 g for IQG5 (IQG5 *vs* CON, *p* < 0.05); and 16.93 ± 5.17 g for CS10 (CS10 *vs* CON, *p* < 0.05) (Fig. 2a). Epididymidal fat was also significantly decreased in the IQG5 and CS10 groups compared to the control: 10.38 ± 2.58 g for control; 8.19 ± 4.00 g for CaG5; 7.00 ± 1.35 g for IQG5 (IQG5 *vs* CON, *p* < 0.05); and 8.56 ± 2.53 g for CS10 (CS10 *vs* CON, *p* < 0.05) (Fig. 2a).

### Adipocyte density

The adipocyte density [number cells/area (mm^2^)] of rat liver tissues treated with GAG in a HFD, as assessed by toluidine blue O staining, were reduced by CaG5 (33.00 ± 1.73), IQG5 (45.00 ± 4.36), or CS10 (26.00 ± 2.52) (CS10 vs CON, *p* < 0.05) when compared to the control (51.00 ± 5.29) (Fig. 2b).

### Blood pressure and heart rate changes

No significant differences in blood pressure (systolic blood pressure or heart rate) were observed between the 5 mg/kg CaG-, IQG-, or CS-treated groups and the control group (data not shown).

### Hematology and blood chemistry

**Fig. 3:**
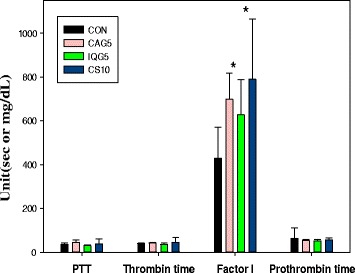
Anticoagulant activity in plasma level of aged rats treated with CaG over a month. CON : PBS (as a vehicle) treated with murine normal diet. Each value represents mean ± S.D. *Statistically significant from the control (*p* < 0.05)

Some dose-dependent changes were observed between the treatment and control groups with respect to the hematological parameters examined at the end of the experiment. There was a 27.5% increase in APTT (sec.) in the CaG5-treated group: CON, 35.68 ± 5.68; CaG5, 45.49 ± 10.91; IQG5, 29.93 ± 3.57; CS10, 36.83 ± 23.95 (Fig. 3). Thrombin time (sec.) was as follows: CON, 40.18 ± 2.04; CaG5, 42.13 ± 3.23; IQG5, 36.15 ± 6.67; CS10, 45.02 ± 21.89. There was a significant (62.6%) increase in Factor I (fibrinogen, mg/dL) in CaG5-treated group: CON, 429.33 ± 142.22; CaG5, 698.29 ± 120.28 (CaG5 *vs* CON, *p* < 0.05); IQG5, 627.14 ± 160.67; CS10, 790.00 ± 274.22 (CS10 *vs* CON, *p* < 0.05). Prothrombin time (sec.) was as follows: CON, 62.86 ± 47.30; CaG5, 52.71 ± 4.96; IQG5, 52.14 ± 5.58; CS10, 55.00 ± 9.92

### Serum biochemistry

In sera from the IQG- and CS-treated groups (Table 1), phospholipid levels (mg/dL) were significantly lower than in control sera after 1 month in aged-rats: CON, 271.78 ± 91.74; CaG5, 233.67 ± 71.0; IQG5, 173.2 ± 32.02 (IQG5 *vs.* CON, *p* < 0.05); CS10, 259.7 ± 57.27 (CS10 *vs.* CON, *p* < 0.05). Free fatty acid levels (μEq/L) were decreased: CON, 686.78 ± 104.67; CaG5, 431.33 ± 66.22 (CaG5 *vs.* CON, *p* < 0.05); IQG5, 405.6 ± 63.39 (IQG5 *vs.* CON, *p* < 0.05); CS10, 566.9 ± 115.38 (CS10 *vs.* CON, *p* < 0.05). Also, serum GPT (ALT) levels (IU/L) in the CaG- and IQG-treated groups were significantly lower than those in the control group in aged rats: CON, 76.33 ± 40.21; CaG5, 36.78 ± 8.0 IU/L; IQG5, 43.22 ± 13.86 (IQG5 *vs.* CON, *p* < 0.05); CS10, 53.44 ± 11.36 (CS10 *vs.* CON, *p* < 0.05). Furthermore, the mean creatinine phosphokinase (CK U/L) level in the CaG-treated group was lower than that in the control group in aged rats: CON, 125.78 ± 91.66; CaG5, 102.89 ± 65.73 (CaG5 *vs.* CON, *p* < 0.05); IQG5, 91.22 ± 24.74; CS10, 131.2 ± 62.56. Mean serum glucose (mg/dL) was significantly decreased in aged rats (each group *vs* CON, *p* < 0.05): CON, 435.11 ± 105.42; CaG5, 304.0 ± 44.44; IQG5, 275.9 ± 43.18; CS10, 317.8 ± 60.31.

**Table 1:** Serological findings of aged rats treated with CaG or IQG for 1 month

Organ function	Parameter	Unit	CON	CaG5	IQG5	CS10
Fatty liver	Phospholipid	mg/dL	271.78 ± 91.74	233.67 ± 71	173.2 ± 32.02*	259.7 ± 57.27*
	Hyaluronic acid(HA)	ng/mL	36.44 ± 12.66	30.44 ± 8.19	35 ± 2.96	42.89 ± 22.06
	Free fatty acid	μEq/L	686.78 ± 104.67	431.33 ± 66.22*	405.6 ± 63.39*	566.9 ± 115.38*
Tonic	Albumin(S)	g/dL	4.33 ± 0.4	3.59 ± 0.34*	3.52 ± 0.29*	3.83 ± 0.36*
Hepatitis	ALP	U/L	85.44 ± 27.06	82.89 ± 19.9	158.9 ± 39.78	99.89 ± 22.24
	AST(SGOT)	U/L	76.67 ± 34.68	52.22 ± 10.3	60.89 ± 5.69	56.33 ± 12.19
	ALT(SGPT)	U/L	76.33 ± 40.21	36.78 ± 8.0*	43.22 ± 13.86*	53.44 ± 11.36*
	LDH	U/L	500.22 ± 403.35	247.22 ± 170	216 ± 60.61	345.9 ± 202.14
Heart Function	CK	U/L	125.78 ± 91.66	102.89 ± 65.73*	91.22 ± 24.74	131.2 ± 62.56
Diabetes	Glucose(S)	mg/dL	435.11 ± 105.42	304 ± 44.44*	275.9 ± 43.18*	317.8 ± 60.31*
Lipidemia	Cholesterol,total	mg/dL	212.67 ± 92.82	181.44 ± 68.4	124.1 ± 23.6*	198.9 ± 44.43
	Triglyceride	mg/dL	207.89 ± 101.95	136.22 ± 61.6	103.3 ± 35.59*	183.4 ± 104.74
	LDL Cholesterol	mg/dL	74.67 ± 44.91	69 ± 31.3	41.44 ± 10.14*	65.22 ± 18.52
	HDL Cholesterol	mg/dL	110.56 ± 10.22	92.22 ± 24.5	78.22 ± 9.40*	114.2 ± 7.85
	Creatinine	mg/dL	0.6 ± 0.09	0.53 ± 0.06	0.55 ± 0.06	0.56 ± 0.05
Nepritis	BUN	mg/dL	19.09 ± 2.45	16.71 ± 3.07	19.36 ± 2.91	20.92 ± 3.27
	Uric acid	mg/dL	10.9 ± 2.26	8.12 ± 1.48*	6.49 ± 0.89*	8.14 ± 2.17*
Edema	Protein,total	mmol/L	7.46 ± 0.32	6.91 ± 0.24*	7.23 ± 0.25	7.23 ± 0.35
Rheumatis	CRP(HS)	mg/L	0.51 ± 0.48	0.32 ± 0.13	0.42 ± 0.24	0.31 ± 0.19

Each value represents mean ± S.D. Asterisk marks (*) mean significant differences compared with the control (PBS) group (*p* < 0.05)

The mean total cholesterol level (mg/dl) in the IQG-treated group was lower than in the control group in aged rats, and that difference was also significant: CON, 212.67 ± 92.82; CaG5, 181.44 ± 68.4; IQG5, 124.1 ± 23.6 (IQG5 *vs.* CON, *p* < 0.05); CS10, 198.9 ± 44.43.

In addition, triglyceride levels (mg/dL) were as follows: CON, 207.89 ± 101.95; CaG5, 136.22 ± 61.6; IQG5, 103.3 ± 35.59 (IQG5 *vs.* CON, *p* < 0.05); CS10, 183.4 ± 104.74. LDL cholesterol (mg/dL) levels decreased: CON, 74.67 ± 44.91; CaG5, 69.0 ± 31.3; IQG5, 41.44 ± 10.14 (IQG5 *vs.* CON, *p* < 0.05); CS10, 65.22 ± 18.52. Significant uric acid level (mg/dL) decreases were seen in all GAG-treated groups as compared with the control (each group *vs.* CON, *p* < 0.05): CON, 10.9 ± 2.26; CaG5, 8.12 ± 1.48; IQG5, 6.49 ± 0.89; CS10, 8.14 ± 2.17, Table 2.

**Table 2:** Antioxidant enzyme activities of dung beetle glycosaminoglycan in aged rat liver

Oxydative enzyme	Unit	CON	CaG5	IQG5	CS10
Catalase	mg protein/min	14.77 ± 2.81	21.18 ± 3.88	15.8 ± 4.55	16.73 ± 3.55
Glutathione peroxidase	Unit/mg protein	2.46 ± 0.15	2.59 ± 0.14^*^	2.48 ± 0.21	2.59 ± 0.1
Glutathione-s-transeferase	nmol/min/ml	3.07 ± 0.09	3.52 ± 0.24^*^	3.04 ± 0.10	3.37 ± 0.07
Superoxide dismutase	nmol/min/ml	381.44 ± 85.32	400.56 ± 60.62	386.72 ± 69.22	513.23 ± 60.07

Each value represents mean ± S.E. Asterisk marks (*) mean significant differences compared with the control (PBS) group (*p* < 0.05)

### Decrease of oxidative damage

**Fig. 4:**
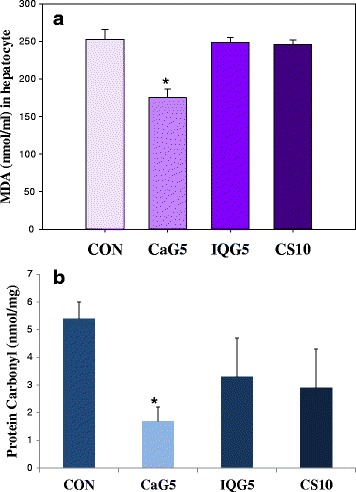
**a** Anti-oxidative effect of CaG on Lipid (MDA) and **b** protein (carbonyl content) after 1-month treatment. Each value represents mean ± S.D. *Statistically significant from the control (*p* < 0.05)

Malondialdehyde (MDA, nmol/ml) was assayed after 1 month of each GAG treatment [CON, 252.9 ± 13.1; CaG5, 175.1 ± 5.8 (30.32% decreases). Each GAG and chondroitin sulfate treatment decreased the lipid peroxidation in hepatocytes (Fig. 4a). The protein carbonyl concentration in blood was decreased at a ratio of 68.52%, 36.89% and 53.70% in GaG5, IQG5 and CS10, respectively (Fig. 4b). But, each GAG had no statistical differences compared with the control in hepatocyte carbonyl content (data not shown).

### Oxidative enzyme (catalase, GPx, GST, SOD) quantitation

Catalase activity (mg protein/min) in hepatocytes after 1 month of GAG treatment was as follows: CON, 17.72 ± 2.81; CaG5, 19.87 ± 3.88; IQG5, 17.22 ± 4.55; CS10, 19.23 ± 3.55. Catalase activity in all CaG-treated hepatocyte groups increased. Super oxide dismutase (SOD) is a free radical (super oxide) scavenging enzyme. SOD activity (nmol/min/ml) increased in the treatment groups as compared with the control: control, 381.44 ± 85.32; CaG5, 400.56 ± 60.62; IQG5, 386.72 ± 69.22; CS10, 513.05 ± 60.07. Glutathione peroxidase activity (unit/mg protein) in the CaG-treated hepatocyte group was significantly increased by the treatment (CaG5 vs. CON, *p* < 0.05), and glutathione s-transferase activity (nmol/min/ml) in the CaG group was also significantly increased compared to that in the control group (CaG5 vs. CON, *p* < 0.05) (Table 2).

### Cytokine IL-1β and IL-10 production

**Fig. 5:**
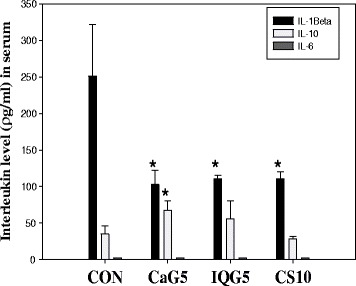
Interleukin level (IL-1beta, IL-6 or IL-10) of aged rats treated with CaG

Increases in IL-10 levels were observed in the CaG-treated group. IL-1β (ρg/ml) levels in serum were reduced by 1 month of CaG or IQG treatment, demonstrating the compounds’ anti-inflammatory actions: control, 251.3 ± 70.7; CaG5, 102.9 ± 9.5; IQG5, 110.4 ± 5.2; CS10, 110.4 ± 10.1 (each group vs. CON, *p* < 0.05). IL-10 activity (ρg/ml) after 1 month of CaG treatment in rats was increased: CON, 34.9 ± 11.5; CaG5, 67.4 ± 13.3 (CaG5 vs. CON, *p* < 0.05); IQG5, 55.5 ± 24.7; CS10, 28.0 ± 3.8 (Fig. 5). The IL-10 levels in the CS10 group, was statistically different from that in the CON group (CS10 *vs*. CON, *p* < 0.05).

### Gene expression by quantitative real-time PCR analysis

The data represented that PCR cycle required number (Ct value) when concentration of PCR amplicon are equilibrated by sample amplicon. Median CT values of 5 sample’s represented, but there was no different from control group (Ct vale >2.0) on used primer sequence (data not shown).

### DNA microarray

Microarray analysis using a Mouse 28 K cDNA clone array was performed in order to identify the gene-expression profiles in CaG, IQG, and CS-treated 15-month-old SD rat livers, and to provide information on potential anti-aging markers.

Compared to the control group, the CaG5 group exhibited a 2- to 4-fold increase in 118 genes, a 4- 8-fold increase in 11 genes, and a greater than 8-fold increase in 2 genes. The expression levels of the remaining ~30,000 genes remained the same. Sixty genes were downregulated from 2- to 4-fold, and 4 showed a 4- to 8-fold decrease.

In the IQG5 group as compared to the control group, 162 genes increased by 2- to 4-fold, 10 genes increased by 4- to 8-fold, and 3 genes increased by more than 8-fold. On the other hand, 66 were downregulated by 2- to 4-fold and 7 were reduced by 4- to 8-fold.

In the CS10 group as compared to the control group 63 genes were upregulated by 2- to 4-fold, 2 genes increased by 4- to 8-fold, and 2 genes were upregulated by more than 8-fold. Thirty-eight genes were downregulated by 2- to 4-fold, 4 were downregulated by 4- to 8-fold, and 4 decreased by more than 8-fold.

When compared to the control group, the lipocalin2 (Lcn2) gene was increased by about 10-fold in CaG5-treated liver tissue, 46-fold in the IQ5 group, and 23-fold in the CS10 group. In liver tissues of CaG5-treated mice, lysil oxidase (Lox) was downregulated at a 0.13 ratio, that is, gene expression was decreased. CaG5 treated rat group, compared to control, showed that 131 genes including lipocalin 2, liposaccharide binding protein (Lbp), and serine peptidase inhibitor, Kaszal type3 (Spink3) were up-regulated (Table 3) and 64 genes including lysyl oxidase (Lox), serine dehydratase (sds) and retinol saturase (Retsat) were down-regulated (Table 4). The CaG5-treated group, compared to the control group, exhibited an upregulation of 131 genes, including lipocalin 2, liposaccharide binding protein (Lbp), and the serine peptidase inhibitor Kaszal type 3 (Spink3) (Table 3) and a downregulation of 64 genes, including lysyl oxidase (Lox), serine dehydratase (sds), and retinol saturase (Retsat) (Table 4). These data point to lipocalin 2 and adipokines, as upregulated genes, and lysyl oxidase (related to heparanase), as a downregulated gene, as potential therapeutic markers that work against aging and obesity

**Table 3:** Upregulated genes differentially expressed in the liver tissue of aged rats

	CaG5	IQG5	CS10	Description	Gene Symbol
1	10.69*	45.83	23.12	lipocalin 2	Lcn2
2	8.15	3.42	1.74	chemokine (C-X-C motif) ligand 1 (melanoma growth stimulating activity, alpha)	Cxcl1
3	6.76	12.07	3.15	S100 calcium binding protein A8	S100a8
4	6.16	3.62	3.61	abhydrolase domain containing 3	Abhd3
5	5.21	3.56	5.38	alpha-2-macroglobulin	A2m
6	4.81	3.17	5.65	myxovirus (influenza virus) resistance 2	Mx2
7	4.75	7.36	9.85	WDNM1 homolog	LOC360228
8	4.45	1.79	1.22	solute carrier family 13 (sodium-dependent citrate transporter), member 5	Slc13a5
9	4.39	6.16	1.85	S100 calcium binding protein A9	S100a9
10	4.36	1.71	1.64	metallothionein 2A	Mt2A
11	4.06	2.23	1.37	kininogen 1-like 1	Kng1l1
12	3.95	2.26	2.35	ATP-binding cassette, sub-family G (WHITE), member 2	Abcg2
13	3.62	1.95	1.85	protocadherin 18	Pcdh18
14	3.52	2.08	1.51	signal-regulatory protein alpha	Sirpa
15	3.50	2.84	2.64	lipopolysaccharide binding protein	Lbp
16	3.46	3.94	3.51	serine peptidase inhibitor, Kazal type 3	Spink3
17	3.36	4.48	2.76	CD36 molecule (thrombospondin receptor)	Cd36
18	3.15	2.92	2.57	serine (or cysteine) peptidase inhibitor, clade A (alpha-1 antiproteinase, antitrypsin), member 7	Serpina7
19	3.11	1.34	2.03	endothelial cell-specific molecule 1	Esm1
20	3.03	2.09	1.03	transglutaminase 1, K polypeptide	Tgm1
21	2.97	1.67	0.84	basic helix-loop-helix family, member a15	Bhlha15
22	2.97	1.70	1.26	growth arrest and DNA-damage-inducible, beta	Gadd45b
23	2.87	2.53	2.44	flavin containing monooxygenase 5	Fmo5
24	2.86	3.52	3.16	serine peptidase inhibitor, Kazal type 3	Spink3
25	2.86	4.39	2.74	cytochrome P450, family 7, subfamily a, polypeptide 1	Cyp7a1
26	2.85	1.75	1.47	phosphoinositide-3-kinase, class 3	Pik3c3
27	2.83	1.57	1.84	solute carrier family 10 (sodium/bile acid cotransporter family), member 2	Slc10a2
28	2.80	1.45	1.62	neuron navigator 2	Nav2
29	2.78	2.68	1.61	stefin A2-like 3	Stfa2l3
30	2.76	1.60	1.01	STEAP family member 4	Steap4
31	2.72	1.90	1.40	desmocollin 2	Dsc2
32	2.71	1.68	1.63	lin-7 homolog a (C. elegans)	Lin7a
33	2.71	2.21	2.01	tumor necrosis factor receptor superfamily, member 21	Tnfrsf21
34	2.71	1.65	1.78	metallothionein 1a	Mt1a
35	2.68	1.32	1.02	interleukin 34	Il34
36	2.66	1.91	1.82	Lanosterol synthase (2,3-oxidosqualene-lanosterol cyclase)	Lss
37	2.66	1.60	1.24	interleukin 1 receptor, type II	Il1r2
38	2.65	1.87	1.61	growth arrest specific 6	Gas6
39	2.64	4.31	1.99	interleukin 1 beta	Il1b
40	2.63	2.04	1.75	sema domain, immunoglobulin domain (Ig), short basic domain, secreted, (semaphorin) 3C	Sema3c

*Corrected for background intensity using local background correction

**Table 4:** Downregulated genes differentially expressed in the liver tissue of aged rats

	CaG5	IQG5	CS10	Description	Gene Symbol
1	0.13	0.35	0.20	lysyl oxidase	Lox
2	0.19	0.53	0.75	similar to Spindlin-like protein 2 (SPIN-2)	LOC367746
3	0.24	0.42	0.90	serine dehydratase	Sds
4	0.28	0.24	0.63	cytokine inducible SH2-containing protein	Cish
5	0.29	0.64	0.65	retinol saturase (all trans retinol 13,14 reductase)	Retsat
6	0.35	0.45	0.62	similar to hypothetical protein MGC42105	RGD1308116
7	0.36	0.28	0.39	solute carrier family 25, member 30	Slc25a30
8	0.36	1.01	0.39	aldo-keto reductase family 1, member B7	Akr1b7
9	0.37	0.19	0.26	one cut homeobox 1	Onecut1
10	0.38	0.66	0.68	CCR4 carbon catabolite repression 4-like (S. cerevisiae)	Ccrn4l
11	0.38	0.62	1.58	patatin-like phospholipase domain containing 3	Pnpla3
12	0.38	0.49	0.95	myotubularin related protein 7	Mtmr7
13	0.39	0.18	0.26	one cut homeobox 1	Onecut1
14	0.40	0.85	1.11	keratin 10	Krt10
15	0.40	0.99	0.85	cytochrome P450, family 17, subfamily a, polypeptide 1	Cyp17a1
16	0.41	0.88	1.07	glutamate receptor, ionotrophic, AMPA 3	Gria3
17	0.41	0.70	0.65	cAMP responsive element modulator	Crem
18	0.41	0.96	1.46	glycerol-3-phosphate acyltransferase, mitochondrial	Gpam
19	0.42	1.00	1.49	sulfotransferase family, cytosolic, 1C, member 2	Sult1c2
20	0.42	0.30	0.44	family with sequence similarity 89, member A	Fam89a
21	0.42	0.32	0.34	solute carrier family 25, member 30	Slc25a30
22	0.42	1.20	0.98	integrin, alpha 1	Itga1
23	0.45	0.73	0.80	glutamic-oxaloacetic transaminase 1, soluble (aspartate aminotransferase 1)	Got1
24	0.45	1.29	1.05	guanine nucleotide binding protein, alpha 14	Gna14
25	0.45	0.37	0.76	similar to Urinary protein 3 precursor (RUP-3)///similar to Urinary protein 2 precursor (RUP-2)	LOC68 0367///LO
26	0.46	0.81	0.74	similar to Maltase-glucoamylase, intestinal	LOC679818
27	0.46	0.63	0.69	ADAM metallopeptidase domain 8	Adam8
28	0.46	0.63	0.74	G0/G1switch 2	G0s2
29	0.46	0.65	0.70	somatostatin receptor 3	Sstr3
30	0.46	0.58	0.69	cAMP responsive element modulator	Crem
31	0.47	0.57	0.56	RRS1 ribosome biogenesis regulator homolog (S. cerevisiae)	Rrs1
32	0.47	0.49	0.50	choline kinase alpha	Chka
33	0.47	0.70	0.86	early growth response 1	Egr1
34	0.48	0.45	0.37	G patch domain containing 4	Gpatch4
35	0.48	1.15	1.11	similar to ovostatin-2	RGD1565709
36	0.48	0.42	0.66	regulator of G-protein signaling 3	Rgs3
37	0.49	0.65	0.51	tsukushin	Tsku
38	0.49	0.75	1.58	mesenchyme homeobox 2	Meox2
39	0.49	0.87	0.71	cyclin D1	Ccnd1
40	0.49	0.39	1.45	phosphoglycerate dehydrogenase	Phgdh

PairMean ratio* means pair Mean ratio (test/control)

### Characterization of CaG

The compositions of the amino, acidic, and neutral monosaccharides of GaG were determined by GC-MS (Table 5). The primary amino monosaccharides of CaG are in the following order: D-glucosaminic acid > N-acetyl-galactosamine > N-acetyl-D-gactosaminitol > galactosamine HCl > galactosaminic acid > galactosamine > glucuronic acid. Also, the neutral monosaccharides found in CaG are mainly α-glucose and mannitol, whereas the minor ones include arabinose and rhamnose.

**Table 5:** Monosaccharide composition of used CaG

Acidic and amino sugar	CaG (μg/mg)	Monosugar (ng/mg)	CaG
D-Glucuronic Acid	8.02	Arabinose	8.79
Glucosamine HCl	10.21	Rhamnose	7.04
Galactosamine HCl	52.17	Ribose	4.72
N-Acetyl-Glucosamine	184.38	Mannose	1.26
D-Glucosaminic Acid	187.96	Galactose	0.92
D- Galactosamic Acid	59.95	α-glucose	13.89
N-Acetyl-DGalactosaminitol	55.50	Mannitol	84.16
Total Sum	558.20	β-glucose	4.12

### Indicators of cardioprotection

**Fig. 6:**
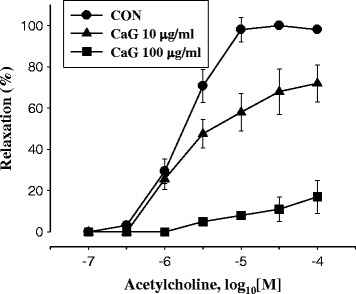
Effect of CaG on endothelium-dependent relaxation in isolated aortic rings from normal rat (*N* = 6). After 1 h-equlibration, aortic rings were treated with vehicle (saline) or Catharsius GAG (10 μg/ml, 100 μg/ml) 10 min before contraction was induced by phenyleprine 1 μM. Then, endothelium-dependent relaxation was induced by acethylcholine (0.1 ~ 100 μM)

In this experiment, we characterized the direct effect of CaG on resistance arteries using vessels incubated *in vitro* with phenylephrine-induced contraction and acetylcholine-induced relaxation. CaG was evaluated as a cardioprotective agent. The concentration-response curve to CaG was shifted downwards compared that seen after control (PBS only) treatment, showing that heart stimulation occurred in a concentration-dependent manner (Fig. 6).

## Discussion

Aging is a process of progressive decline in the physiological capacity of an organism, manifested by accumulated alterations and destabilization at the whole system level [13]. Glycosaminoglycan is thought to diminish the deleterious effects of aging by preventing the destruction of cartilage, bone, discs, skin [23], the vascular system [8], etc. Recently, A type of GAG as a multifunctional materials, heparin base polyurethane or are considered currently as one of the established biocompatible and blood compatible biomaterials offering tremendous structure-property relationship such as a heparin immobilization on chitosan modified polyurethane grafts with anti-adhesive and antimicrobial properties [24]. Nowadays, the advent of temperature control has made possible the design of large-scale insect rearing systems. Therefore, glycosaminoglycan can be acquired from the insect cortex as an alcohol extract residues. Such crude drugs from dung beetles and *B. ignitus* queens have been studied in an effort to identify their active components or to make the extracts safer for human use. The dung beetle *C. molossus* was purified to yield N-acetyldopamine dimers, molossusamide A-C (1–3), showing COX-1 and COX-2 inhibitory activity [25]; chitosan [26]; melanin [27]; and serine proteases [9, 28]. A bumblebee (*Bombus ignitus*) extract was shown to contain bee venom serine protease [29], peroxiredoxins [30], and peptidoglycan recognition protein (PGRP-S) [31].

Strategies to prevent or delay aging have included ingestion of antioxidants for repairing oxidative cellular damage to DNA, proteins, and lipids, as well as stem cell-mediated tissue regeneration and gene therapy [32]. As a lipid oxidative damage marker in hepatocyte lipid oxidative stress states, malondialdehyde was decreased by CaG treatment. Also, carbonyl content in blood, especially neutrophil, was decreased by CAG treatment and showed repairing cellular oxidative protein damage.

Glycosaminoglycans from *C. molossus* and *B. ignitus* queens consist mainly of D-glucosaminic acid as an acidic monosaccharide and N-acetyl-galactosaminitol as an amino monosaccharide, along with α-glucose and D-mannitol as neutral monosaccharides. Thus, the compound can access cell membrane receptors and can prevent the causes of age-related diseases by altering energy metabolism. Glycosaminoglycans could repair connective tissues [23], eroded cartilage, discs, bone, and glycoproteins at the molecular level, with GAG forming a bond between proteins [9]. In this experiment, the rats used were old and weighed about 680 g, so, it would not have been easy to bring about a reduction in body weight, but we did observe diminished adipose fat weight and adipocyte numbers. As with most glycosaminoglycans, CaG and IQG brought about a reduction in serum levels of inflammation-related parameters: free fatty acid, AST (SGPT), creatinine kinase (related heart function), glucose, uric acid, and hyperglycemia related levels: cholesterol, total, triglyceride, LDL cholesterol, etc. The free fatty acid levels (related fatty liver) of the treated groups were statistically significantly decreased, 36.7% in the CaG group and 40.94% in the IQG group. Also, the sGPT level (related liver function) was significantly reduced, 51.58% in the CaG group and 43.38% in the IQG group.

Data generated from the DNA microarray analysis supported the anti-aging effect of IQG, with meaningful changes in gene expression profiles in 14-month-old SD rats observed after a 1-month treatment period. The CaG5-treated rats, compared to the control group, had increases in 131 genes, including lipocalin 2 [33], liposaccharide binding protein (Lbp) [34], and a serine peptidase inhibitor, Kazal type3 (Spink3) [35] and decreases in 64 genes, including lysyl oxidase (Lox) [36], serine dehydratase (sds) [37], and retinol saturase (Retsat) [38]. The data point to lipocalin 2 and adipokines, which were up-regulated, and lysyl oxidase (related to heparanase), which was down-regulated, as potential therapeutic markers for anti-aging effects in obese animals.

The present study observed meaningful changes in gene expression profiles in aged rats after treatment with CaG or IQG for 1 month. The gene expression of lipocalin-2, which is an adipokine implicated in insulin resistance [39] and a secretory protein with lipid-binding prosperities, was increased by about 10 times. This is in agreement with a microarray study that reported that ingestion of soybeans causes an increase of an endogenous amyloid-β chaperone, lipocalin-type prostaglandin D2 synthase (Ptgds), leading to suppression of amyloid-β and preventing cognitive dysfunction [40]. Lipopolysaccharide (LPS) binding protein (LBP), a surrogate marker of microbial translocation, is associated with reduced physical function and increased inflammation [41]. The normal aging process alters blood coagulation system in humans; Natural anticoagulants, including antithrombin III, heparin cofactor II, protein C, protein S, and tissue factor pathway inhibitor, can modulate the reactions of blood coagulation system [42]. In this study, there were significant increases in factor I (fibrinogen) in CaG (162.6%), IQG (146.0%), and CS10 (184.0%)—treated groups when compared to the control, PBS-treated group. Also, the level of LBP (LPS binding protein) was upregulated in our DNA microarray data, suggesting that heparin (GAG) binds to LBP, facilitates the transfer of LPS to CD14, and enhances LPS-mediated activation of peripheral blood monocytes, resulting in anticoagulant activity [43]. A trypsin inhibitor, serine protease inhibitor Kazal-type 3 (Spink3), was upregulated in this experiment, indicating a role for paracrine modulation; it is also involved in embryo implantation [44]. As Spink3 is an important serine protease inhibitor, its upregulation may reflect an important endogenous cytoprotective mechanism that would prevent further injury [45]. The downregulation of lysyl oxidase may indicate that heparin (GAG) inhibiting the tight binding of lysyl oxidase to pre-formed fibrils was hardly affected [46]. The downregulation of retinol saturase seen in this experiment is notable, as retinol saturase promotes adipogenesis and is downregulated in obesity [47]. According to a hypothesis, adipose tissue density, a novel biomarker predicting mortality risk in older adult’s that does not appear to be inflammation related [48], furthermore, CaG reduced significantly adipose density promising potent anti-aging agent.

## Conclusions

Dung beetle glycosaminoglycan decreased cholesterol and triglyceride levels in serum, caused a decrease in the weight of adipose tissue and a normalization of serum levels of free fatty acid, GPT, glucose, and uric acid, and a prolongation of coagulation time that would prevent blood aggregation and lipid accumulation in vascular endothelial barriers that contribute to homeostasis in the circulatory system. IL-1β (pg/ml) in serum was reduced by 1 month of CaG or IQG treatment, demonstrating anti-inflammatory action. In addition, the liver protective gene lipocalin was highly (10-fold) upregulated and lysyl oxidase was downregulated (0.1-fold) in CaG-treated rats. CaG derived from insects could be a safer replacement for heparin and other GAGs from mammalian sources, as insect sources reduce the possibility of transmission of infectious viruses from animals such as pigs. These results suggest that CaG and BIQ could not only be natural anti-aging agents but also be functional foods, similar to chitosan and future biomaterials playing vital role our to-day to day life.

## Abbreviations

ALP: Alkaline phosphatase
ALT(GPT): Glutamate pyruvate transaminase
aPTT: Activated partial thromboplastin time
AST(GOT): Glutamate oxaloacetate transaminase
BUN: Blood urea nitrogen
CaG 5: Dung beetle (*Catharsius molossus*) glycosaminoglycan 5 mg/kg
CK: Creatinine phosphokinase
CON: Control group
CRP: C-reactive protein
CS10: Chondroitin sulfate (10 mg/kg)
GAG: Glycosaminoglycan
GGT: γ-glutamyl transferase
H. Chol: High cholesterol
IQG5: Queen of *Bombus ignitus* (a type of bumblebee) glycosaminoglycan 5 mg/kg
l.Chol: Low cholesterol
LDH: Lactate dehydrogenase
PT: Prothrombin time
T. Chol: Total cholesterol
TG: Triglyceride
